# Studies of Antiviral Activity and Cytotoxicity of *Wrightia tinctoria* and *Morinda citrifolia*

**DOI:** 10.4103/0250-474X.59550

**Published:** 2009

**Authors:** P. Selvam, N. Murugesh, M. Witvrouw, E. Keyaerts, J. Neyts

**Affiliations:** Arulmigu Kalasalingam College of Pharmacy, Anand Nagar, Krishnankoil, 626 190, India; 1 Institute of Pharmacology, Madurai Medical College, Madurai-625 020, India; 2 Molecular Medicine, Katholieke Universiteit-Leuven and IRC Kulak Kapucijnenvoer 33, Leuven B-3000, Flanders, Belgium; 3 Raga Institute for Medical Research, Katholieke Universiteit-Leuven Minderbroederstraat 10, Leuven B-3000, Belgium

**Keywords:** AntiHIV activity, antiHCV activity, *Morinda citrifolia*, *Wrightia tinctoria*

## Abstract

Different extracts of leaf parts of *Wrightia tinctoria* and fruit powder of *Morinda citrifolia* have been studied against replication of HIV-1(IIIB) in MT-4 cells and HCV in Huh 5.2 cells. Chloroform extract of *Wrightia tinctoria* exhibited a maximum protection of 48% against the cytopathic effect of HIV-1(IIIB) in MT-4 cells. Fruit juice of *Morinda citrifolia* exhibited a displayed marked cytotoxic activity in lymphocyte (MT-4) cells (CC50: 0.19 mg/ml). The 50% effective concentration for inhibition of HCV subgenomic replicon replication in Huh 5-2 cells by *Morinda citrifolia* was 0.98 μg/ml and by chloroform extract of *Wrightia tinctoria* was 10 μg/ml. The concentration that reduced the growth of exponentially proliferating Huh 5-2 cells by 50% was greater than 50 μg/ml.

Acquired immunodeficiency syndrome (AIDS) is a life threatening and debilitating disease state caused by retrovirus infection and the etiologic agent is now widely known as the human immunodeficiency virus type-1. Hepatitis C Virus (HCV) causes jaundice, hepatocellular carcinoma and important viral co-infection in HIV/AIDS. Many compounds of plant origin have been identified that inhibit different stages in the replication cycle of HIV[1–4]. *Wrightia tinctoria* is an important medicinal plant used in the Indian system of medicine for the treatment of variety of diseases[5] and it to possessed analgesic[6], cytotoxic[7] activities. *Morinda citrifolia* L (Noni) has been used in folk remedies by Polynesians for over 2000 years and is reported to have a broad range of therapeutic effects[8], including antibacterial, antiviral, antifungal, antitumor, analgesic, hypotensive, antiinflammatory, and immune enhancing effects. The present study is designed to find the antiviral activity of different extracts of *Wrightia tinctoria* (WT) and *Morinda citrifolia* (MC) against the replication of HIV-1(III_B_) and Hepatitis C virus (HCV). The cytotoxicity of WT and MC is also tested against mock-infected MT-4 cells and Huh 5.2 cells.

Leaf parts of WT (Apocynaceae) were collected in and around Tenkasi, Tamilnadu, India and were dried in shade, subjected to hot continuous percolation using ether, chloroform, methanol, ethanol and water. The ether (EWT), chloroform (CWT), methanol (MWT) and ethanol (ETWT) and aqueous extracts (AWT) of WT were concentrated by distillation and used for screening. Fruit juice and powder of MC was a gift from health India laboratory, Chennai, Tamilnadu, India and the fruit powder of MC was subjected to hot continuous percolation using methanol and ethanol. The methanol (MMC) and ethanol (EMC) extract of MC were concentrated by distillation and used for screening.

Extracts of WT and MC were tested for their inhibitory effects against the replication of HIV-1(III_B_) in MT-4 cells[9,10]. The MT-4 cells were grown and maintained in RPMI 1640 Medium supplemented with 10% (v/v) heat-inactivated Fetal Calf Serum (FCS), 2 mM-glutamine, 0.1% sodium bicarbonate and 20 mg/ml gentamicin (culture medium). Inhibitory effect of extracts on HIV-1 replication was monitored by inhibition of virus-induced cytopathic effect in MT-4 cells and was estimated by MTT assay. Briefly, 50 μl of HIV-1(100-300 CCID_50_) were added to a flat-bottomed microtiter tray with 50 μl of medium containing various concentrations of extracts of WT. MT-4 cells were added at a final concentration of 6×10^5^ cells/ml. After 5 days of incubation at 37°, the number of viable cells were determined by the 3-(4,5-dimethylthiazol-2-yl)-2,5-diphenyl tetrazolium bromide (MTT) method. Cytotoxicity of WT against mock-infected MT-4 cells was also assessed by the MTT method. The anti-HIV data are presented in Table 1.

**TABLE 1:** ANTIHIV ACTIVITY AND CYTOTOXICITY OF *WRIGHTIA TINCTORIA* AND *MORINDA CITRIFOLIA*

Compound	EC_50_^a^ (μg/ml)	CC_50_^b^ (μg/ml)	Maximum % Protection
CWT	>50	>50	48
MWT	>44.8	44.8	2
EWT	>50	>50	1
ETWT	>50	>50	2
AWT	>141.24	141.24	1
MC	>0.19	0.19	18
EMC	>72.34	72.34	0
MMC	>220	220	0
AZT(STD)	0.0062	65.45	106

a Concentrations required to inhibit the cytopathic effect of HIV-1(IIIB) in MT-4 cells by 50%.

b Concentrations required to cause cytotoxicity to 50% of the MT-4 cells

Antiviral activity against HCV in Huh 5-3 cells was undertaken with Huh-5-2 cells[10–12] (a cell line with a persistent HCV replication 1389luc-ubi-neo/NS3-3/5.1; replication with firefly luciferase-lubquitin-neomycine phosphotransferase fusion protein EMCV-IRES driven NS3-5B HCV polyprotein) was cultured in RPMI medium 2 mM glutamine, 1× non essential amino acid (Life Technologies, Washington DC); 100 IU/ml penicillin and 100 μg/ml streptomycin and 250 μg/ml G418 (Geneticin, Life Technologies, Washington DC). Cells were seeded at a density of 7000 cells per well in 96 well view plate TM (Packard, CA) in medium containing the same compounds as described above, except for G418. Cells were allowed to adhere and proliferate for 24 h. At that time, culture was removed and serial dilution of test compounds were added in culture medium lacking G418. Interferon α2a (500 IU) was added as a positive control. Plates were further incubated at 37° and 5% CO_2_ for 72 h replication of HCV replication in Huh-5 cells results in luciferase activity in the cells. Luciferase activity was measured by adding 50 μl of 1× Glo-lysis buffer (Promega) for 15 min of followed by adding 50 μl Steady-Glo Luciferase assay reagent (promega). Luciferase activity was measured with a luminometer and signal in each individual well was expressed as a percentage of the untreated culture. Parallel culture of Huh 5-2 cells, seeded at a density of 7000 cells/well of classical 96-well cell culture plates (Becton-Dickenson) were treated in a similar fashion except that no Glo-lysis buffer or Stady-Glo Luciferase reagent was added. Instead the density of the culture was measured by means of the MTS method (Promega). The antiviral activity and cytotoxicity of the extracts are present in Table 2.

**TABLE 2:** ANTIHCV ACTIVITY AND CYTOTOXICITY OF *WRIGHTIA TINCTORIA* AND *MORINDA CITRIFOLIA*

Compound	EC50 (μg/ml) HCV RNA*	EC50 (μg/ml) Cell growth**	SI
CWT	10	>50	>5
MWT	>50	>50	0
EWT	23	>50	>2
ETWT	29	>50	2
MC	0.98	>50	>50

* (% untreted control)

** Interferon alfa-2b at 10.000 units/well reduced the signal in the viral RNA (luciferase) assay to background levels; without any cytostatic activity.

The different extracts of WT and MC have been evaluated for antiHIV activity and cytotoxicity (Table 1) against HIV-1(III_B_) replication in acutely infected MT-4 cells. None of the extracts exhibited antiHIV activity in acutely infected MT-4 cells. CWT exhibited a maximum protection of 48% of the MT-4 cells against the cytopathic effect of HIV-1(III_B_) at subtoxic concentration. However the MWT showed some cytotoxic activity in lymphocyte (MT-4) cells (CC_50_: 44.8 μg/ml). MC exhibited a maximum protection of 18% of the MT-4 cells against the cytopathic effect of HIV-1(III_B_) after acute infection. MC displayed marked cytotoxic activity in lymphocyte (MT-4) cells (CC_50:_ 0.19 μg/ml). Both EMC and MMC displayed cytotoxic activity (CC_50_) in lymphocyte (MT-4) cells at 72.34 and 220 μg/ml, respectively.

The results of antiHCV activity (Table 2) revealed that, 50% effective concentration for inhibition of HCV subgenomic replicon replication in Huh 5-2 cells (luciferase assay) by CWT was 10 μg/ml, whereas EWT and ETWT inhibits HCV RNA synthesis at the concentration (EC_50_) of 23 and 29 μg/ml, respectively. The concentration of extracts reduced the growth of exponentially proliferating Huh 5-2 cells by 50% was greater than 50 μg/ml. MC inhibits the HCV RNA at the concentration of 0.98 μg/ml and cytotoxicity was found to be greater than 50 μg/ml.
